# Effects of Four Weeks of In-Season Pre-Workout Supplementation on Performance, Body Composition, Muscle Damage, and Health-Related Markers in Basketball Players: A Randomized Controlled Study

**DOI:** 10.3390/jfmk9020085

**Published:** 2024-05-10

**Authors:** Athanasios Douligeris, Spyridon Methenitis, Antonios Stavropoulos-Kalinoglou, George Panayiotou, Paris Vogazianos, Antonia Lazou, Konstantinos Feidantsis, Constantinos Giaginis, Konstantinos Papanikolaou, Giannis Arnaoutis, Yannis Manios, Athanasios Z. Jamurtas, Sousana K. Papadopoulou

**Affiliations:** 1 Department of Nutrition Sciences and Dietetics, Faculty of Health Sciences, International Hellenic University, GR-57400 Thessaloniki, Greece; douligeris.a@gmail.com (A.D.); smetheni@phed.uoa.gr (S.M.); kfeidant@upatras.gr (K.F.); 2 Sports Performance Laboratory, School of Physical Education & Sports Science, National and Kapodistrian University of Athens, GR-15772 Athens, Greece; 3 Theseus, Physical Medicine and Rehabilitation Center, GR-17671 Athens, Greece; 4 Carnegie School of Sports, Leeds Beckett University, Leeds LS1 3HE, UK; a.stavropoulos@leedsbeckett.ac.uk; 5 Department of Physical Education & Sport Science, University of Thessaly, GR-42100 Trikala, Greece; kpapanikolaou@uth.gr (K.P.); ajamurt@uth.gr (A.Z.J.); 6 Department of Life Sciences, School of Sciences, European University Cyprus, 1516 Nicosia, Cyprus; g.panayiotou@euc.ac.cy; 7 Department of Social and Behavioral Sciences, School of Humanities, Social and Education Sciences, European University Cyprus, 2404 Nicosia, Cyprus; p.vogazianos@euc.ac.cy; 8 Department of Medicine, School of Health Sciences, National and Kapodistrian University of Athens, GR-11527 Athens, Greece; anlazou@med.uoa.gr; 9 Department of Fisheries and Aquaculture, School of Agricultural Sciences, University of Patras, GR-26504 Mesolonghi, Greece; 10 Department of Food Science and Nutrition, School of Environment, University of the Aegean, GR-81400 Myrina, Greece; cgiaginis@aegean.gr; 11 Department of Nutrition and Dietetics, School of Health Sciences and Education, Harokopio University, GR-17671 Athens, Greece; garn@hua.gr (G.A.); manios@hua.gr (Y.M.); 12 Institute of Agri-Food and Life Sciences, Hellenic Mediterranean University Research Centre, GR-71410 Heraklion, Greece

**Keywords:** long-term effect, aerobic performance, anaerobic performance, agility, countermovement jump, pre-workout, speed, metabolic health, nutritional supplementation, human participants

## Abstract

This randomized, double-blinded, experimental study investigated the effects of a four-week daily pre-workout supplementation (200 mg caffeine, 3.3 g creatine monohydrate, 3.2 g β-alanine, 6 g citrulline malate, and 5 g BCAA) vs. placebo (isocaloric maltodextrin) on anaerobic (jumping, sprinting, agility, and the running-based anaerobic sprint test: RAST) and aerobic (Yo-Yo intermittent recovery test level 1) performance, as well as on body composition and selective muscle damage/health-related blood markers in well-trained basketball players during the in-season period. Eighteen basketball players (age: 24.4 ± 6.3 years, height: 185.7 ± 8.0 cm, weight: 85.7 ± 12.8 kg, body fat: 16.5 ± 4.2%) were randomly assigned into two groups: pre-workout supplement (PWS, *n* = 10) or placebo (PL, *n* = 8). PWS consumption increased aerobic performance (PWS: 8 ± 6%; PL: −2 ± 6%; *p* = 0.004) compared to PL. A significant decrease was observed in peak (F = 7.0; *p* = 0.017), average (F = 10.7; *p* = 0.005), and minimum power (F = 5.1; *p* = 0.039) following 4 weeks of supplementation in both groups. No other significant changes were observed between groups (*p* > 0.05). In conclusion, the consumption of the current PWS over a four-week period appears to positively influence the aerobic performance of well-trained basketball players during the in-season period. However, it does not appear to mitigate the observed decline in anaerobic power, nor does it affect performance in jumping, sprinting, and agility, or alter body composition or selective muscle damage/health-related blood markers.

## 1. Introduction

Basketball is classified as an intermittent-type mixed aerobic and anaerobic activities sport [1]. In particular, since it includes short bursts of high-intensity activities (sprinting, quick changes of direction, acceleration, deceleration, shuffling, backward running, and jumping) followed by short bursts of low-intensity activity (walking, jogging, and short periods of recovery) [2], it is characterized by high requirements of anaerobic/aerobic capacities [3]. Furthermore, highly skilled basketball players are characterized by an increased capacity for speed, strength, and power [4,5,6]. Alongside basketball players’ specific training routines to increase or maintain their performance, several nutritional supplements are used with conflicting results [7].

Recently pre-workout supplements (PWS) have attracted the attention of athletes, coaches, and sports/nutrition scientists because they are thought to improve athletes’ performance [8,9,10]. PWSs are nutrition formulas consumed prior to exercise [10,11], and they usually contain a mixture of ingredients such as caffeine, β-alanine, creatine, nitric oxide agents (e.g., citrulline malate), and amino acids [e.g., branched-chain amino acids (BCAAs)] [12]. The key ingredients prioritized when selecting a pre-workout supplement include caffeine (80.3%), beta-alanine (57.9%), creatine (46.1%), vasodilators (43.6%), and BCAAs (3.4%), with the belief that these substances will increase energy and focus, enhance muscular strength, mass, and endurance, promote blood flow, and facilitate fat loss [13]. Although each ingredient has been individually investigated regarding its beneficial effects on athletes’ performance [14,15,16,17,18], their synergistic effect, when consumed together [19], has not been extensively investigated. Until now, it has been reported that the acute use (i.e., single dose, 30–60 min prior to training) of several PWS types may lead to short-term/acute increases in power, agility, and anaerobic performance in recreationally-trained or resistance-trained participants or athletes [10,20,21,22]. However, other investigations failed to exhibit such a beneficial effect of PWSs on performance [11], probably due to differences in PWS formulation. Unfortunately, most studies investigating the effect of PWSs on human body performance are limited to acute assessments, mainly in untrained or novice/recreationally trained individuals, while the data on athletes, especially professional, well-trained athletes, are extremely scarce. From the limited available data on basketball players, it seems that the acute use of PWSs exhibited improvements in anaerobic power [10,22] and agility performance while sprinting [10], and aerobic performance seems not to be affected [10,22]. 

Presently, only scarce data exists regarding the effect of long-term PWS consumption in team-sports athletes, especially during the in-season period, when athletes have several weeks of frequent games and a limited amount of time for specific strength and conditioning training sessions. Currently, most of the long-term studies have focused on endurance sports. PWS consumption for ten weeks appeared to induce beneficial changes in elite cyclists’ anaerobic and aerobic performance [23]. In contrast, shorter periods of PWS consumption (e.g., four weeks) are ineffective for improvements in strength, anaerobic, and aerobic performances in endurance runners [24,25]. However, due to the lack of studies on team sports, the effects of long-term PWS consumption on various aspects of athletes’ performance, body composition, and muscle damage/health blood markers, particularly during the in-season period, are yet to be conclusively determined [1,26,27,28]. The latter is particularly important since coaches face several issues, such as match-induced fatigue (mostly related to the accumulated workload from consecutive weekly matches), athletes’ weekly training schedules, recovery, and preparation between matches [1,26,27,28]. Consequently, the training routines, which aim to increase/maintain strength, power, aerobic, and anaerobic performances, are limited or minimized during the in-season periods [1,26,27,28]. Therefore, this period could be ideal for long-term PWS consumption, as it may lead to the maintenance or even the improvement of basketball players’ specific performances and decrease muscle damage and fatigue, since several of the commonly included ingredients in PWS have been proposed as evidence-based post-exercise/training recovery strategies [7,27].

Considering all the above, the present study aimed to investigate the effect of a four-week PWS protocol on jumping, sprinting, agility, anaerobic, and aerobic performances, body composition, and muscle damage/health-related blood markers in basketball players during the in-season period. It was assumed that a four-week PWS consumption should improve basketball players’ specific performance and decrease muscle damage and fatigue without changing their body composition.

## 2. Materials and Methods

### 2.1. Study Design

**Figure 1:**
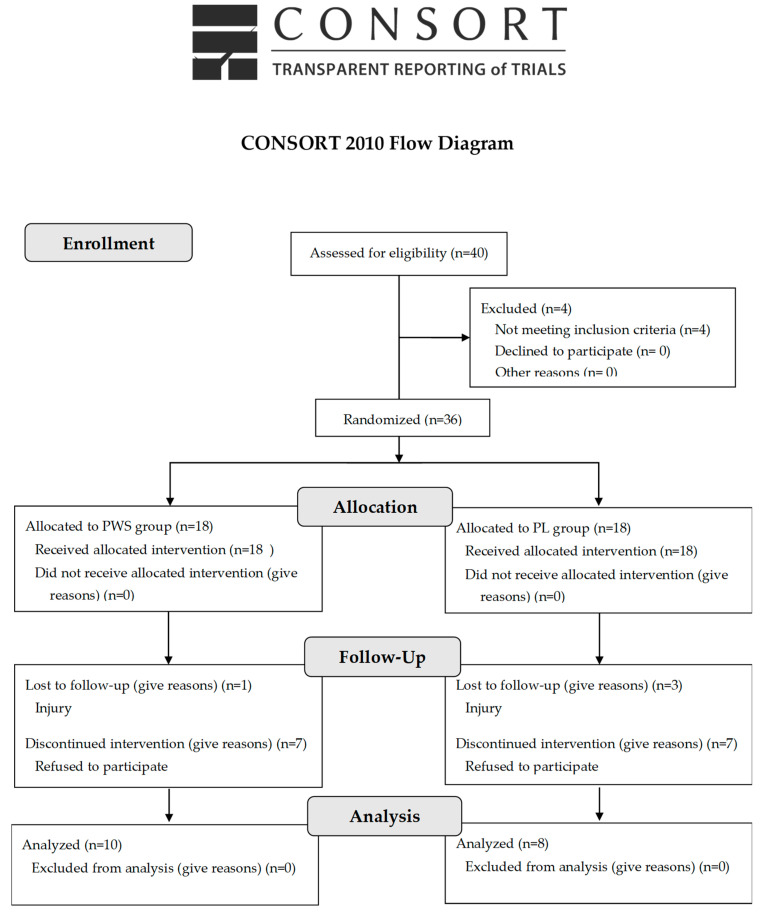
The consolidated standards of reporting trials (CONSORT) diagram of the study.

The study employed a randomized, double-blind, placebo-controlled experimental design. Participants were randomly assigned to one of two groups (PWS vs. placebo (PL)), utilizing block randomization [29] to mitigate reporting bias. Figure 1 shows the CONSORT diagram of the study. All procedures related to this study were in accordance with the Helsinki Declaration and approved by the International Hellenic University’s ethics committee for the protection of human participants (ref. number 07/09.06.2022), and the study was registered at ClinicalTrials.gov with the identifier NCT06059911.

The present study took place during the in-season period in which all athletes followed their regular weekly training program, consisting of five days of training and one game per week without any significant changes in frequency, volume, or intensity of training. Recruitment occurred among male athletes from local basketball teams competing in the Athens first-division championship in Greece. Inclusion criteria comprised (1) male gender, (2) minimum of five training days per week for at least one year, (3) absence of orthopedic/neuromuscular issues, (4) no caffeine hypersensitivity [as assessed by the health history questionnaire (HHQ)], (5) consumption of no more than three servings of coffee daily, (6) no prior use of PWSs or steroids, (7) no consumption of creatine, β-alanine, citrulline malate, or BCAA-protein supplementation for at least six months prior to the study, (8) no use of drugs, abuse or medications, that are known to affect basketball-specific anaerobic and aerobic performance, and (9) consent to adhere to a standard dietary regimen for basketball players.

Participants meeting the inclusion criteria visited our lab (first visit) for medical screening, signed a written informed consent, and completed a 3-day nutritional recall questionnaire before measurements initiation, following detailed explanations from our registered nutritionists. They were also instructed to maintain the same diet for three days prior to subsequent performance tests. Four days prior to fitness evaluations, participants were reminded to replicate the 3-day nutritional recall questionnaire and confirm that they had followed it. During the second visit, participants were familiarized with the performance measurements at submaximal effort. Before the initiation of performance tests and on all occasions, participants underwent a standardized 15 min warm-up comprising low-intensity running and dynamic lower-body stretching. The baseline performance evaluations occurred one week later during the third visit. Participants were advised to refrain from alcohol or caffeine consumption and exercise for at least 24 h prior to physical performance evaluation. Participants’ blood samples were collected before the initiation of performance evaluations. The measurements were conducted following (order of evaluation and methodologies) the National Strength and Conditioning Association (NSCA) standards [2] and were: (1) anthropometric measurements (body height, mass, and composition) and vital signs (resting-heart-rate blood pressure and heart rate); (2) counter-movement jump (CMJ); (3) 20 m sprint; (4) agility T-test; (5) running-based anaerobic sprint test (RAST), and (6) Yo-Yo intermittent recovery test level 1 (Yo-Yo IRL1 $\dot{V}$O_2max_). A 30 min rest interval separated the evaluation of RAST and Yo-Yo IRL1, while a 10 min rest period was observed between all other assessments. After completing the baseline evaluations, a research team member administered the PWS or PL supplements to the participants based on their group allocation. They were instructed to consume one dose daily for the following four weeks, 30 min before training, and upon waking up on off days, and they all consented. The fourth visit occurred after the end of the four-week intervention period, wherein participants underwent the same evaluations as in the baseline visit in the same order. 

### 2.2. Participants

Eighteen active, well-trained basketball players (age: 24.4 ± 6.3 years; training sessions per week: 6 ± 0.6; training session duration: 1.5 ± 0.1 h; resistance training sessions per week: 1 ± 1.8; and resistance training duration 0.5 ± 0.6 h) volunteered to participate and complete the present study. The participants were divided into two groups: the PWS group and the PL group.

### 2.3. Performance Tests and Measurements

#### 2.3.1. Anthropometry and Resting Vital Signs

Height was measured using a stadiometer (Leicester portable height measure, Tanita HR 001, Tokyo, Japan), while body mass, body-fat percentage, body-fat mass, and fat-free mass were assessed using a calibrated digital scale and bioelectrical impedance analysis (Tanita BF-522W, Tokyo, Japan). Blood pressure and heart rate were measured using an upper-body blood pressure digital monitor (Omron M2 Basic, Kyoto, Japan).

#### 2.3.2. Jumping Performance

Counter-movement jump (CMJ) performance was evaluated using a contact mat (CM 60 × 43, ALGE-TIMING, Lustenau, Austria) according to established protocols [30,31,32,33]. After a standard warm-up, participants performed three submaximal CMJs. After 3 min of rest, participants performed three maximal attempts (with hands akimbo), with 3 min of rest between them. The best performance was retained for analysis. The CMJ test has demonstrated a high reliability coefficient of 0.98 [34].

#### 2.3.3. Sprint Performance

The study evaluated the time required to complete a 20 m sprint on a basketball court, following established methodologies [30,31,32,33]. Photocell gates (Photocell PR1aW, ALGE-TIMING, Lustenau, Austria, accuracy of 0.01 s) positioned 0.4 m above the ground were utilized to accurately record timing, with reflectors placed 1 m apart at the start (0 m) and finish (20 m) lines. Participants underwent three submaximal sprints, interspersed with three minutes of rest between each. Subsequently, participants completed three maximal sprints from a standing start position, with 3 min intervals between attempts [30,31,32,33]. The best performance from the maximal sprints was retained for statistical analysis (ICC = 0.91).

#### 2.3.4. Agility T-Test (ATT)

Agility, a critical skill for basketball players [35], has been recognized as a physiological prerequisite for successful performance in basketball [2]. Agility was assessed using the ATT, comprising four multidirectional, basketball-specific movements: sprinting, bidirectional lateral shuffling, and backward running. This test evaluates athletes’ ability to quickly change direction and assesses overall agility [36,37]. For the purpose of this study, a standard protocol outlined for ATT was implemented [38,39]. The participants completed three ATT routines on a wooden basketball court, with a standardized rest period of five minutes between each trial. Test times were recorded using photocell gates (Photocell PR1aW, ALGE-TIMING, Lustenau, AT, accuracy of 0.01 s), and the best performance was further utilized for statistics (ICC: 0.98).

#### 2.3.5. Running-Based Anaerobic Sprint Test (RAST)

RAST served as a reliable measure of basketball players’ anaerobic capacity [40], exhibiting a test reliability coefficient of 0.88 [41]. The basketball-specific RAST protocol involved six maximal 35 m round-trip runs, interspersed with two 17.5 m shuttle runs and 10 s rest intervals [42]. Photocell gates (Photocell PR1aW, ALGE-TIMING, Lustenau, Austria, precision of 0.01 s) positioned 0.4 m above the ground accurately determined sprint timing. Post-test, various power variables were calculated, including power = weight (kg) × distance (m^2^) ÷ time (s^3^). Maximum power = the highest value of six sprints; minimum power = the lowest value of six sprints; average power = sum of all six values ÷ 6; and fatigue index (FI) = (peak power − minimum power/peak power) × 100. The ICC for these evaluations ranged from 0.89 to 0.94.

#### 2.3.6. Yo-Yo Intermittent Recovery Test Level 1

The Yo-Yo intermittent recovery test level 1 (Yo-Yo IRL1) served as a valuable tool for assessing aerobic capacity [36] in the field, with test–retest reliability ranging from 0.78 to 0.98 [43]. The Yo-Yo IRL1 $\dot{V}$O_2max_ test comprised multiple 40 m stages of progressively increased speed, with participants required to sprint 20 m, turn, and return to the starting line according to controlled audio signals. Between stages, athletes have a 10 s period of active recovery (2 × 5 m of jogging). The test commenced at 10 km/h, with speed increments of 0.5 km/h per round, and ended when the participant voluntarily ceased or failed to complete shuttle runs in time on two consecutive occasions due to increased fatigue [44]. The total distance covered during the test was used to calculate maximum oxygen consumption ($\dot{V}$O_2max_). $\dot{V}$O_2max_ was estimated using the following specific equation: $\dot{V}$O_2max_ (mL/min/kg) = IR1 distance (m) × 0.0084 + 36.4 [44].

#### 2.3.7. Supplementation

The PWS developed for this study (BIONATIV SRL, Bucharest, Romania) comprised 200 mg caffeine (2.4 ± 0.4 mg/kg of body weight), 3.3 g creatine monohydrate, 3.2 g β-alanine, 6 g citrulline malate, and 5 g BCAA per dose, aligning with recommended effective dose ranges (caffeine: 3–6 mg/kg body mass, creatine: 3–5 g/day, β-alanine: 4–6 g/day, citrulline malate: 6–8 g/day, BCAA: 5–10 g/d) [45,46,47,48,49]. Moreover, the PWS remained under 100 kcal/serving to avoid any gastrointestinal issues during exercise. Participants in the PWS group consumed one scoop (20 g, ~73 Kcal, tropical fruit flavor) of powder diluted in 300 mL water for four weeks. The PL group consumed one scoop (20 g, ~74 Kcal, 97% flavored maltodextrin, similar in color, flavor, taste, and energy as PWS) of powder diluted in 300 mL of water over the same four-week period. To ensure a double-blind approach, a study member who was not involved in any data collection shared both supplement and placebo jars in all participants, including a 20 g scoop. Participants received PWS or PL supplements 30 min before their training session or after waking up on off days. Every day, a research member contacted all participants to remind them to take their daily dose of PWS or PL until the end of the supplementation period to ensure that all participants received either the PWS or PL supplements, depending on their group.

#### 2.3.8. Blood Sampling and Biochemical Assays

Venous blood samples were obtained from each subject after overnight fasting prior to and after the supplementation period. Blood samples were drawn in Vacutainer-type tubes containing a clot activator to determine biochemical parameters and then centrifuged for 30 min. The recovered sera were stored at −40 °C until further analysis for various biochemical parameters, including lactate dehydrogenase (LDH), creatine kinase (CPK), alkaline phosphatase (ALP), γ-glutamyl transferase (γ-GT), urea, creatinine, glutamic-oxaloacetic transaminase (SGOT), and glutamic-pyruvic transaminase (SGPT). All samples were analyzed by an external laboratory (Abbot Laboratories Architect ci8200 Chemistry Analyzer; P. Mantouvalou Diagnostic Laboratory AE, Athens, Greece).

### 2.4. Statistical Analyses

A post hoc power analysis (G*Power ver. 3.1; FrankFaul, Universitat Kiel, Kiel, Germany) was performed according to the study design, the number of participants, and the mean difference in Yo-Yo IRL1 $\dot{V}$O_2max_, which revealed an actual power of 0.91, an effect size of 0.66, and an alpha criterion of 0.05 for the results of the present study. A Shapiro–Wilk test was used to assess the normality of the data. Normally distributed data are presented as means ± SD and non-normally distributed data as median (interquartile range) [50]. A two-way repeated-measures ANOVA (2 × 2; time [pre vs. post] × group [PWS vs. PL]) was used for free-fat mass, resting diastolic blood pressure, resting heart rate, RAST test, Yo-Yo IRL1 $\dot{V}$O_2max_, LDH, CPK, ALP, γ-GT, urea, SGOT and SGPT. Non-parametric Mann–Whitney U analysis was used for body weight, body fat, fat mass, resting systolic blood pressure, counter-movement jump, 20 m sprint, agility T-test, and creatinine. Independent sample T-tests were used for the comparison of the percent change from pre- to post-values in each variable between the groups. Statistical analyses were performed with SPSS Statistics Version 29, while the significance was set at *p*  <  0.05. 

## 3. Results

No significant differences were observed between the PWS and PL groups in any of the variables at baseline evaluation (*p* > 0.05). No significant changes were observed in body mass, body fat, free-fat mass, or resting BP and HR between groups from pre- to post-evaluation (*p* > 0.05; Table 1).

**Figure 2:**
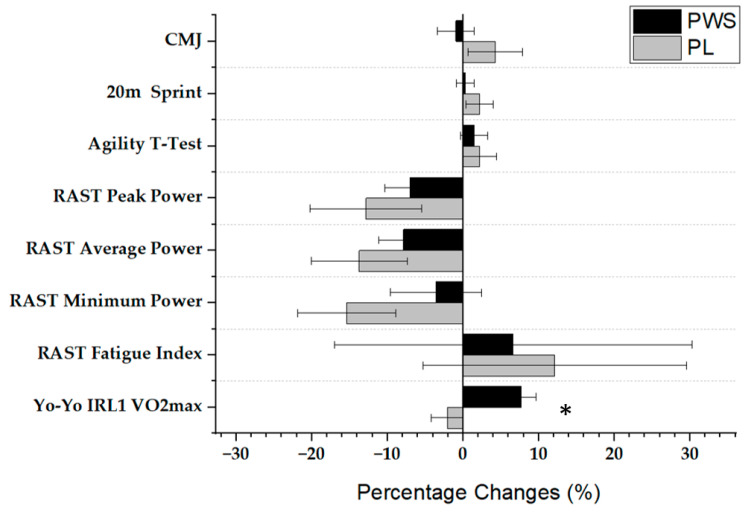
Pre- to post-intervention percentage changes (%) in basketball performance measurements. Asterisk (*) denotes statistically significant differences (*p* < 0.05) between PWS and PL groups in each parameter.

A significant time [pre vs. post]–group [PWS vs. PL] interaction was observed in aerobic performance (F = 12.1; *p* = 0.003) (Table 2). Aerobic performance increased significantly more in the PWS group compared to the PL group (%Δ: PWS: 7.7 ± 6.3; PL: −2.1 ± 6.0; *p* = 0.004) (Figure 2). A significant change was observed in time [pre vs. post] in the anaerobic peak (F = 7.0; *p* = 0.017), average (F = 10.7; *p* = 0.005), and minimum power (F = 5.1; *p* = 0.039) in both groups, whereas no significant time–group interaction was observed (*p* > 0.05) (Table 2). Moreover, the PWS group did not significantly prevent the observed deterioration in anaerobic peak (%Δ: PWS: 22127 ± 10%; PL: −13 ± 21%; *p* > 0.05), average (%Δ: PWS: −8 ± 10%; PL: −14 ± 18%; *p* > 0.05), and minimum power (%Δ: PWS: −4 ± 19%; PL: −15 ± 18%; *p* > 0.05), compared to the PL (Figure 2). No significant changes were observed in CMJ, 20 m sprint, agility T-test, or FI, from pre- to post-values within or between groups (*p* > 0.05; Table 2; Figure 2).

**Figure 3:**
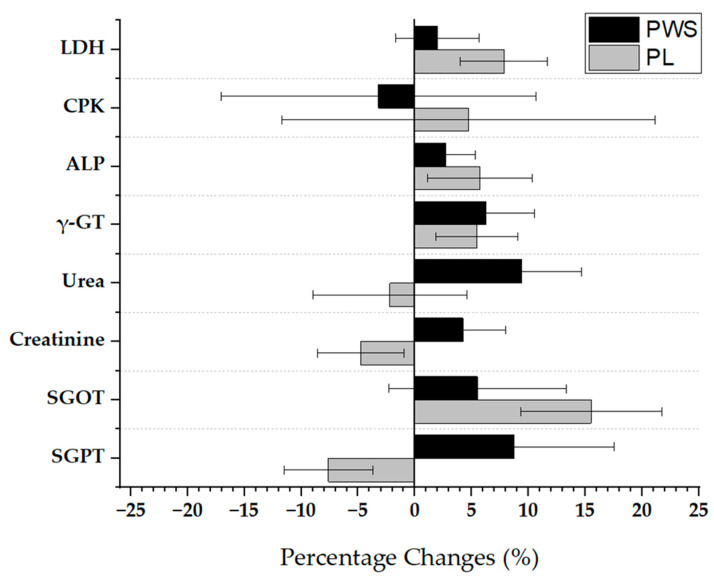
Pre- to post-intervention percentage changes (%) in metabolic health indices with error bars.

No significant changes were also observed in any of the evaluated muscle damage/health-related blood markers within or between the PWS and PL groups at any time point (*p* > 0.05; Table 3; Figure 3).

## 4. Discussion

The present study aimed to assess the effect of four-week pre-workout supplement (PWS) consumption on jumping, sprinting, agility, anaerobic and aerobic performance indices, body composition, and muscle damage/health-related markers in basketball players during the in-season period. The main findings were that four weeks of PWS supplementation improved basketball players’ aerobic performance but had no significant effect on other performance indices, body composition, or muscle damage/health-related markers.

### 4.1. Aerobic Performance

To our knowledge, this is the first study to evaluate the long-term PWS effectiveness on aerobic performance in basketball players. Improvements in aerobic performance were found after the four-week intervention in the PWS group compared to PL. During the study protocol, all athletes in both groups followed the same/similar training routines, and thus, it is unlikely that the observed increases in the aerobic capacity of the PWS’s athletes can be attributed to their training routines. The improvement in aerobic performance indices is probably attributed to the caffeine, creatine, β-alanine, citrulline malate, and BCAA consumption included in this PWS. Caffeine intake (3–6 mg/kg/day) seems to decrease carbohydrate use during exercise and improves endurance-exercise capacity by 2–4% [51]. Chronic creatine supplementation increases work and aerobic capacity through greater ATP transfer from mitochondria [52]. Chronic β-alanine supplementation within the range of 2–6 g/day may decrease skeletal muscles’ acidosis during exercise, which results in lower neuromuscular fatigue, as well as improvements in aerobic performance and time to exhaustion (TTE), at least in recreationally active participants [53,54]. Moreover, when creatine is combined with β-alanine for 28 days (5.25 g creatine and 1.6 g β-alanine), it may also improve endurance performance [55], underscoring the synergistic action of the two substances when consumed together. L-citrulline supplementation for more than seven days in dosages of 2.4–6 g/day increases nitric oxide (NO) bioavailability, potentially leading to beneficial increases in aerobic performance [56,57]. L-citrulline supplementation may also delay fatigue during high-intensity exercise through improved ammonia removal and decreased lactic acid formation in the blood [58]. Furthermore, citrulline coupling with malate for fifteen days may further improve performance, enhance oxidative energy production, and decrease the sensation of fatigue [59], since their synergistic action is involved in ammonia detoxification through the urea cycle, attenuation of lactate production, and enhancement of aerobic pyruvate utilization [47]. Lastly, BCAA supplementation (20 g) before exercise may increase BCAA oxidation, decrease the uptake of free tryptophan by the brain, decrease serotonin synthesis, and, consequently, delay central fatigue during exercise and, thus, lead to improved time to exhaustion (TTE) in long-distance runners [60]. The present study’s findings are consistent with the data of Fernández-Lázaro et al. [23], who reported improvements in aerobic performance after a long-term, ten-week PWS consumption in elite-cyclists who benefited from a similar dosage range of substances. Specifically, the doses of citrulline malate (6 g), creatine (5 g), and β-alanine (4 g) used in the study of Fernández-Lázaro et al. [23] were similar to the amount of citrulline malate (6 g), creatine (3.3 g), and β-alanine (3.2 g) of the present study’s PWS. Considering all the above, it seems that, when these substances are consumed together and within the effective dosage ranges [46,47,52], improvements in aerobic performance may be exhibited.

### 4.2. Anaerobic Performance

In the present study, anaerobic power values were decreased in both groups. Despite the fact that the magnitude of the decrease in anaerobic power values was lower in the PWS group compared to the PL, no significant changes were observed between groups after the post-supplementation period. The observed decreases in anaerobic power could be attributed to the accumulated fatigue of basketball players from frequent, exhausting matches and training sessions, as the study was conducted during the late stages of the in-season period [61]. Although it was expected that the intervention period would lead to enhancements in anaerobic power, attributed to certain ingredients found in this PWS, such as creatine, β-alanine, and/or BCAAs, which been reported to improve anaerobic performance [46,52,62], no such effect was observed. Ingredients such as creatine and β-alanine supplementation may exert a favorable effect on anaerobic performance after long-term consumption [46,52]. More specifically, chronic creatine supplementation has been suggested for intermittent sports athletes (like basketball players), since high-intensity repeated exercise performance is typically increased by 10% to 20% depending on muscle PCr enhancement following creatine loading [52]. BCAA consumption could also enhance anaerobic performance, since short-term (seven days of 5 g/day) supplementation seems to improve anaerobic performance in football players during the RAST test [62]. On the other hand, long-term consumption of caffeine or citrulline malate does not seem to contribute to anaerobic performance improvements, since limited evidence exists [47,51]. However, even if, according to the above, an increase in the anaerobic performance of PWS’s athletes was expected, this was not found in the present study. In similar intervention studies, Köhne et al. [24], Ormsbee et al. [25], and Hoffman et al. [63] found no effect on anaerobic power in endurance-trained runners or healthy college students after 28 days of PWS consumption. In contrast, Fernández-Lázaro et al. [23] showed that a longer (ten weeks) PWS consumption period significantly improved anaerobic power in elite cyclists. This disparity is most likely attributed to differences in the study protocols, which suggest that a longer supplementation period (ten weeks) could be more effective than a shorter one (four weeks) for improvements in anaerobic power. However, this remains to be investigated in future studies.

### 4.3. Jumping, Sprinting, and Agility Performance

No significant changes were observed in jumping, sprinting, or agility performance between the PWS or PL groups. It should be highlighted that the effectiveness of long-term PWS consumption on speed and agility in basketball players has never been examined so far. Improvements were expected to be reported in the present study since creatine’s and/or β-alanine’s chronic consumption could improve athletes’ performance during short-term high-intensity exercise. More specifically, creatine chronic supplementation (3–5 g/d) increases PCr resynthesis, decreases muscle acidosis, and can enhance acute exercise capacity by allowing team-sports athletes, such as basketball players, to perform better over a series of sprints [52]. A four-week β-alanine supplementation (4–6 g/d) increases skeletal muscle carnosine levels, acting as a hydrogen ions (H^+)^ buffer regulator that attenuates neuromuscular fatigue and improves performance in high-intensity short-duration exercise [46]. However, in the present study, no improvements were observed after a 4-week PWS consumption. Kohn et al. [24] also reported no improvements in jumping height after four weeks of supplementation using a similar PWS containing caffeine, creatine, β-alanine, and BCAA. We can assume that the reason for these observations may depend on the length of our intervention, since an extended supplementation period could be more effective for improvements [23]. Another possible reason could be that the present study took place during the in-season period, since athletes’ fatigue from in-season high training and match demands could inhibit any potential beneficial effect of the present PWS. 

### 4.4. Body Mass and Body Composition

The results of the present study reported no significant changes between the PWS or PL group for body mass and body composition. Moreover, no improvements were observed in body mass or composition, despite the presence of potentially effective ergogenic substances that were included in the used PWS. Creatine, in a daily dosage range of 3–5 g, can effectively increase phosphocreatine levels over 3–4 weeks, leading to greater training adaptations and gains in lean mass [52]. Additionally, a minimum four-week loading phase of 4 g of β-alanine is crucial for increasing muscle carnosine levels [46] and improving lean mass [64]. Therefore, the duration of the intervention may have been insufficient to allow the ergogenic effects of the above substances to take place. The current study’s results are consistent with the data of Smith et al. [65], who found no differences in body composition following a 3-week PWS consumption during high-intensity exercise. Conversely, other studies investigating the effect of prolonged PWS consumption on body composition have exhibited a favorable effect, since PWS supplementation in combination with a resistance training regimen leads to improvements in free-fat mass following six to eight weeks of supplementation [66,67]. Thus, future studies with longer supplementation periods will determine the possible effectiveness of PWS on muscle mass and body composition in basketball players during the in-season period. 

### 4.5. Muscle Damage/Health-Related Blood Markers

The safety of isolated PWS components like creatine [68], β-alanine [69], citrulline [47], caffeine [70], and amino acids [71] has been well investigated. Some ingredients included in the present study on PWSs, such as creatine and BCAA, have been found to decrease muscle damage markers. More specifically, creatine supplementation may lead to a decrease in LDH [72] or CPK [72,73] muscle damage markers’ levels, probably through the amelioration in the inflammatory response and oxidative stress, regulation of calcium homeostasis, and activation of satellite cells [74]. In a recent meta-analysis, BCAA supplementation benefits the muscle damage marker CPK but not LDH [18]. In contrast, β-alanine supplementation seems to have no significant effect on exercise-induced CPK and LDH markers following four weeks of supplementation in female basketball players [75]. Similarly, citrulline malate, following seven days of supplementation, did not improve CPK and LDH markers in football players [76], and caffeine supplementation seems insufficient for improvements in CPK levels [77]. To our knowledge, the present study is the first to assess muscle damage/health-related blood markers in basketball players following a 4-week PWS consumption. Although improvements were expected in muscle damage markers, no significant differences were found in CPK and LDH muscle damage markers, probably due to the short duration of the supplementation protocol (4 weeks) since a longer supplementation period (10 weeks) seems to be more beneficial for improvements in athletes’ muscle damage markers [23]. It is worth mentioning that although CPK levels exceeded the reference interval in both groups during baseline measurements, minimal and non-significant changes were observed after the PWS supplementation period. The present study’s findings also suggest that a 4-week PWS consumption could not lead to abnormal changes in health-related markers, such as ALP, γ-GT, urea, creatinine, SGOT, and SGPT, since no significant effect was observed. These data are in accordance with the findings of previous studies that assessed PWS safety for four weeks and reported that PWS consumption for four weeks remains safe in recreationally trained participants [78,79]. Most of the ingredients contained in the PWS and used for analysis in these studies, such as caffeine, creatine, β-alanine, citrulline malate, or BCAA [78,79], were similar to the PWS employed in the present study. 

### 4.6. Side Effects and Vital Signs

The athletes observed or reported no significant side effects (e.g., gastrointestinal issues, diarrhea, cramps) during the study. However, in two athletes of the PWS group, a tingling sensation in body parts, such as the neck and the backside of the hands, was observed. The latter (lasting approximately 60 min) could be attributed to high doses of β-alanine consumption, with no potential danger to the athletes according to the literature [46]. Finally, as expected, no significant changes were observed after four weeks of PWS consumption, even if it includes several substrates, such as caffeine and β-alanine, that have been reported to affect cardiovascular vital signs (heart rate and blood pressure). The results of the present study verified those of a previous study investigating the acute effect of a PWS on cardiovascular vital signs, reporting that PWS does not affect them [10]. Thus, considering the above, it can be suggested that long-term use of a PWS supplementation containing 200 mg caffeine, 3.3 g creatine monohydrate, 3.2 g β-alanine, 6 g citrulline malate, and 5 g BCAA per dose is safe enough to be used by well-trained basketball players during the in-season period.

### 4.7. Study’s Limitations

The first limitation of this study was that we did not investigate each ingredient’s long-term effect separately on basketball performance indices. The second limitation was that we assessed the effect of PWS on muscle damage/health-related markers only for four weeks. Therefore, the safety of PWS supplementation for three months, six months, or one year cannot be determined. Moreover, parameters such as sleep patterns and heterogeneity, due to the inclusion of subjects from different teams, may have influenced the outcomes measured. An additional limitation is the lack of an objective method to verify whether athletes had indeed consumed the PWS for at least 6 months prior to the study, which could be addressed in future investigations. Finally, the present study examined only the long-term effects of this PWS supplementation on anaerobic and aerobic performance indices. Given that strength is a crucial factor in basketball players’ performance, future studies are expected to investigate the longitudinal effect of such a PWS on basketball players’ strength. Moreover, future studies may benefit from implementing stricter control measures and considering more homogeneous participant samples to better elucidate the observed effects.

## 5. Conclusions

**Figure 4:**
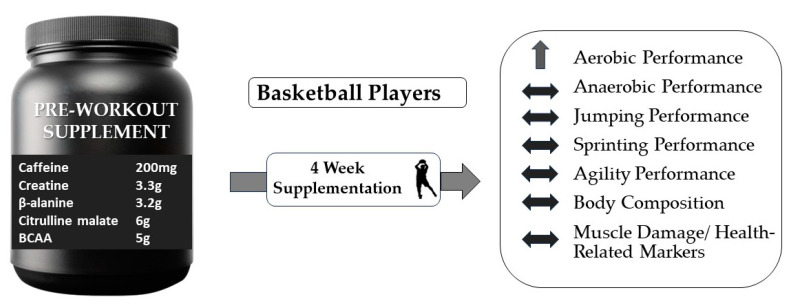
A schematic representation of the study innovation and key findings.

In conclusion, the present investigation suggests that four weeks of PWS consumption (containing 200 mg caffeine, 3.3 g creatine monohydrate, 3.2 g β-alanine, 6 g citrulline malate, and 5 g BCAA per dose) may improve aerobic performance in basketball players during the in-season period. However, this supplement seems insufficient for improvements in jumping, sprinting agility, or anaerobic power. Moreover, four weeks of PWS intervention could not significantly affect body composition or muscle damage/health-related bloodborne markers in basketball players (Figure 4). Further investigation into various sports, specifically team sports, will determine in the future the effectiveness of these products on athletes’ performance.

## Figures and Tables

**Table 1:** Anthropometric data and vital signs pre- and post-4 weeks of pre-workout (PWS) or placebo (PL) supplementation.

	Pre-Workout Supplement (PWS) (*n* = 10)		Placebo (PL) (*n* = 8)	
	Pre	Post	Pre	Post
Height (cm)	184.2 ± 9.4	184.2 ± 9.4	187.5 ± 5.9	187.5 ± 5.9
Body Weight (kg)	78.6 (20.8)	78.7 (20.3)	84.4 (13.7)	84.2 (13.4)
Body Fat (%)	15.5 (6.7)	14.7 (6.1)	15.9 (4.5)	15.0 (4.8)
Free-Fat Mass (kg)	70.2 ± 9.6	70.8 ± 9.5	72.3 ± 4.8	72.8 ± 5.1
Fat-Mass (kg)	12.7 (7.3)	12.4 (6.4)	13.1 (5.6)	13.1 (5.7)
Resting Systolic Blood Pressure (mmHg)	132 (9)	132 (4)	136 (10)	130 (17)
Resting Diastolic Blood Pressure (mmHg)	75 ± 11	75 ± 9	79 ± 6	77 ± 8
Resting Heart Rate (beats/min)	63.8 ± 8.1	65.2 ± 6.4	62.9 ± 5.8	64.5 ± 5.4

Data presented as mean ± SD or median (interquartile range).

**Table 2:** Performance indices pre- and post-4 weeks of pre-workout (PWS) or placebo (PL) supplementation.

	PWS (*n* = 10)		PL (*n* = 8)	
	Pre	Post	Pre	Post
Counter-Movement Jump Height (cm)	41.0 (11.0)	41.5 (9.0)	37.5 (6.0)	39.0 (11.0)
20 m Sprint (s)	3.05 (0.21)	3.04 (0.19)	3.25 (0.49)	3.23 (0.61)
Agility T-Test (s)	10.48 (0.75)	10.52 (0.77)	10.62 (0.82)	10.54 (1.65)
RAST Peak Power (watt)	358.4 ± 48.0	331.4 ± 47.9 ^#^	336.8 ± 49.6	278.3 ± 54.5 ^#^
RAST Average Power (watt)	308.4 ± 41.6	281.8 ± 34.7 ^#^	282.3 ± 27.0	247.4 ± 52.2 ^#^
RAST Minimum Power (watt)	255.9 ± 46.7	241.5 ± 42.2 ^#^	254.5 ± 37.4	212.3 ± 43.2 ^#^
RAST Fatigue Index, FI (%)	28.3 ± 10.1	24.2 ± 9.4	24.0 ± 8.1	25.7 ± 11.5
$\mathrm{Yo}\text{-}\mathrm{Yo}\;\mathrm{IRL}1\;\dot{V}$O_2max_ (mL·kg^−1^·min^−1^)	43.9 ± 3.3	47.3 ± 4.1 *	44.3 ± 3.4	43.2 ± 2.1

Data presented as mean ± SD or median (interquartile range). (*) denotes significant differences between groups in the marked time point; (^#^) denotes significant differences within groups from pre- to post-values (*p* < 0.05).

**Table 3:** Muscle damage and metabolic health markers pre- and post-four weeks of pre-workout (PWS) or placebo (PL) supplementation.

	PWS (*n* = 10)		PL (*n* = 8)		Reference Interval
	Pre	Post	Pre	Post	
LDH (U/L)	186 ± 27	188 ± 29	182 ± 15	196 ± 22	125–220
CPK (U/L)	236 ± 86	217 ± 89	215 ± 63	252 ± 32	30–180
ALP (U/L)	73 ± 15	75 ± 12	70 ± 14	72 ± 9	38–126
γ- glutamyl transferase (γ-GT) (U/L)	16 ± 3	17 ± 4	16 ± 4	17 ± 4	11–50
Urea (mg/dL)	33 ± 7	35 ± 6	36 ± 10	35 ± 8	10–50
Creatinine (mg/dL)	0.9 (0.1)	1.0 (0.2)	1.1 (0.3)	1.0 (0.2)	0.5–1.5
SGOT (U/L)	27 ± 8	28 ± 9	25 ± 3	27 ± 4	0–46
SGPT (U/L)	24 ± 7	25 ± 6	23 ± 7	24 ± 8	0–46

Data presented as mean ± SD or median (interquartile range).

## Data Availability

The data presented in this study are available from the corresponding author upon reasonable request.
